# Consistency of the Empirical Distributions of Navigation Positioning System Errors with Theoretical Distributions—Comparative Analysis of the DGPS and EGNOS Systems in the Years 2006 and 2014

**DOI:** 10.3390/s21010031

**Published:** 2020-12-23

**Authors:** Mariusz Specht

**Affiliations:** Department of Transport and Logistics, Gdynia Maritime University, Morska 81-87, 81-225 Gdynia, Poland; m.specht@wn.umg.edu.pl

**Keywords:** statistical distribution, navigation positioning system, position error, Differential Global Positioning System (DGPS), European Geostationary Navigation Overlay Service (EGNOS)

## Abstract

Positioning systems are used to determine position coordinates in navigation (air, land and marine). The accuracy of an object’s position is described by the position error and a statistical analysis can determine its measures, which usually include: Root Mean Square (RMS), twice the Distance Root Mean Square (2DRMS), Circular Error Probable (CEP) and Spherical Probable Error (SEP). It is commonly assumed in navigation that position errors are random and that their distribution are consistent with the normal distribution. This assumption is based on the popularity of the Gauss distribution in science, the simplicity of calculating RMS values for 68% and 95% probabilities, as well as the intuitive perception of randomness in the statistics which this distribution reflects. It should be noted, however, that the necessary conditions for a random variable to be normally distributed include the independence of measurements and identical conditions of their realisation, which is not the case in the iterative method of determining successive positions, the filtration of coordinates or the dependence of the position error on meteorological conditions. In the preface to this publication, examples are provided which indicate that position errors in some navigation systems may not be consistent with the normal distribution. The subsequent section describes basic statistical tests for assessing the fit between the empirical and theoretical distributions (Anderson-Darling, chi-square and Kolmogorov-Smirnov). Next, statistical tests of the position error distributions of very long Differential Global Positioning System (DGPS) and European Geostationary Navigation Overlay Service (EGNOS) campaigns from different years (2006 and 2014) were performed with the number of measurements per session being 900’000 fixes. In addition, the paper discusses selected statistical distributions that fit the empirical measurement results better than the normal distribution. Research has shown that normal distribution is not the optimal statistical distribution to describe position errors of navigation systems. The distributions that describe navigation positioning system errors more accurately include: beta, gamma, logistic and lognormal distributions.

## 1. Introduction

**Figure 1:**
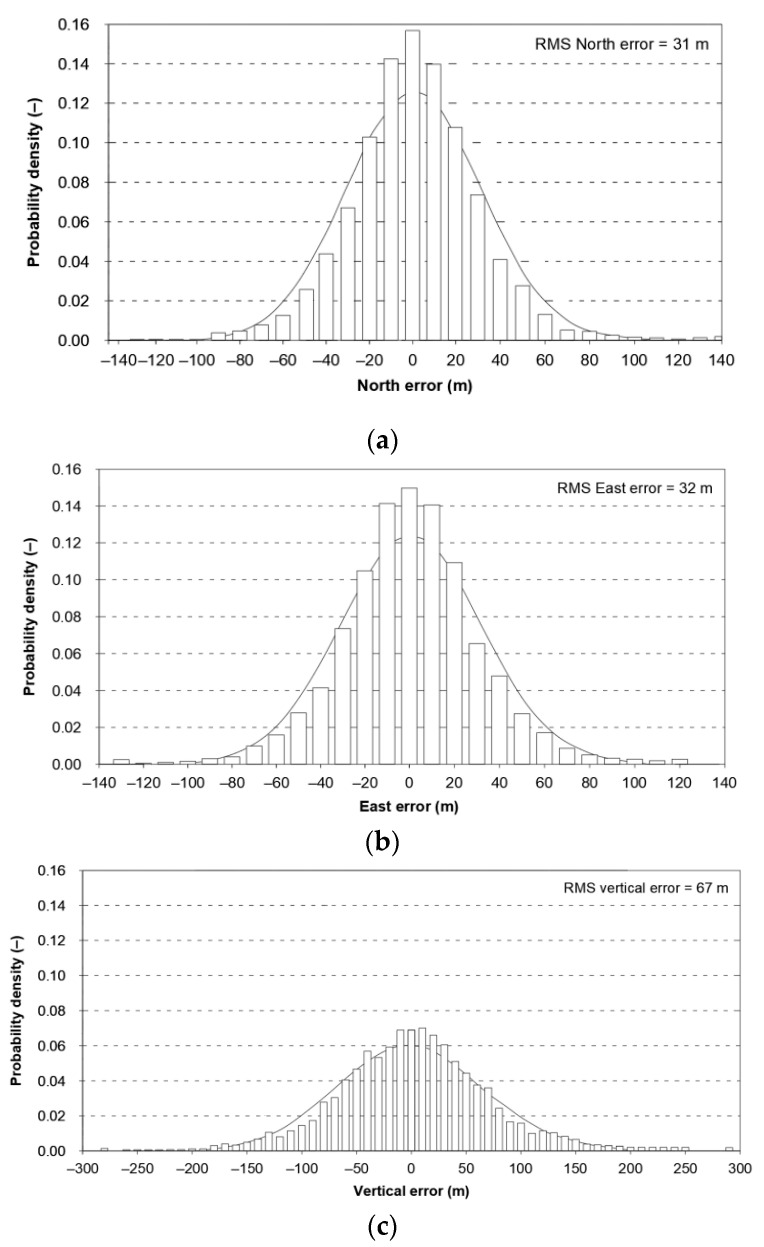
Comparison of empirical data of the GPS position error in North (**a**), East (**b**) and vertical (**c**) axes with the theoretical normal distribution. Own study based on: [6].

The assumption that a position line error in navigation has a normal distribution is commonplace for book authors [1,2,3], as well as in monographs, regulations and standards directly related to the statistical analysis of position errors [4,5]. It should be noted, however, that several scientific publications draw attention to existing differences between the empirical and theoretical distributions. Global Positioning System (GPS) is the basic positioning system used in navigation. Its operational characteristics are periodically described in several technical standards, of which Global Positioning System Standard Positioning Service Signal Specification versions have already been issued five times; in 1993, 1995, 2001, 2008, and 2020. The first version of this document [6] states expressly that the empirical error distributions are overlaid with Gauss distributions, as a basis for comparison with theoretical expectations (Figure 1). The theoretical distributions were generated using the means and standard deviations of the empirical datasets. The error distributions are based upon measured data from the GPS Control Segment monitor stations recorded for three months.

**Figure 2:**
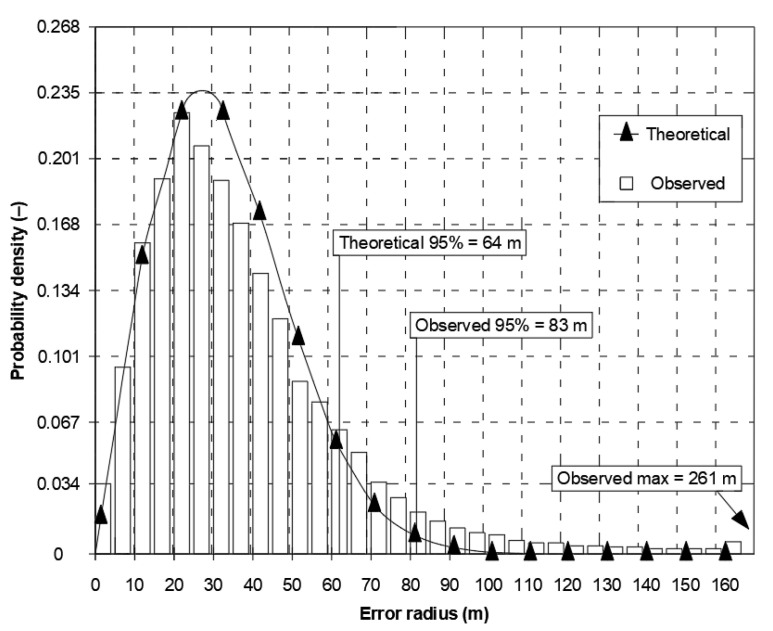
Nominal GPS SPS horizontal error distribution. Own study based on: [6].

The presented differences in local axes must result in significant differences in the fit of 2D position error with the chi-square distribution. Therefore, in the same document, Figure 2 presents the empirical (64 m) and theoretical (83 m) values of twice the Distance Root Mean Square (2DRMS) measure. It should be stressed that since the estimation error was as high as 19 m, 95% of the measurements will be smaller than this. In this situation, it is difficult to support the use of normal distribution for the calculation of the basic quantity describing the system positioning accuracy (2DRMS). Similar conclusions concerning the inconsistency of the statistical distributions of Differential Global Positioning System (DGPS) and GPS position errors are raised by the Frank van Diggelen, but with much smaller discrepancies [5].

In the authors’ research on the accuracy of various navigation positioning systems, two measures of accuracy were also often compared: 2DRMS and R95. The latter is an empirical quantity calculated by sorting errors from the smallest to the largest. This value is higher than 95% of the measurements made. Please note that if the empirical 2DRMS statistics fit the chi-square distribution, these values should be almost identical. The author’s research into the accuracy of various Global Navigation Satellite Systems (GNSS), such as DGPS and European Geostationary Navigation Overlay Service (EGNOS) [7], GNSS geodetic networks, as well as multi-GNSS solutions [8,9], has repeatedly shown significant discrepancies between 2DRMS and R95 measures.

In order to quantify the discrepancy between the 2DRMS and R95 measures, let us analyse the results of the position accuracy tests of six different mobile phones working in parallel, which were conducted in 2017. The same smartphones were tested during both dynamic [8] and stationary [9] measurement campaigns. To compare the fit of both values, the concept of Relative Percent Error (RPE) has been introduced, according to the relationship:

(1) $$RPE=\frac{R95\left(2D\right)-2DRMS\left(2D\right)}{R95\left(2D\right)}\cdot 100\%$$

The obtained results are presented in the last rows of Table 1 and Table 2.

The research results indicated that the differences between the values of 2DRMS and R95 may reach a dozen percent or so. Therefore, it can be assumed that there may be significant differences between empirical distributions of latitude (*φ*) and longitude (*λ*) errors and the normal distribution. This problem may concern various navigation positioning systems, so it is justified to undertake more research into testing the actual results obtained by positioning systems.

This article examines the statistical fit between empirical distributions with theoretical position errors of two Differential Global Navigation Satellite Systems (DGNSS): marine DGPS and EGNOS. Two measurement campaigns of both systems were used for research purposes, during which more than 1–2 million fixes were recorded. Since there numerous measurements, the conclusions drawn from them can be considered representative. The research were carried out in the years 2006 and 2014.

The aim of the publication is:

- Determining the consistency of empirical distributions with the theoretical (normal and chi-square) for DGPS and EGNOS position errors. Latitude and longitude errors were referred to as the normal distribution and 2D position errors were referred to as the chi-square distribution.
- Finding statistical distributions other than normal and chi-square distributions that present a better fit with DGPS and EGNOS empirical data.
- Comparison of the statistical distributions of DGPS and EGNOS position errors from 2006 and 2014 will make it possible to answer the following question: do the statistical distributions of 1D and 2D position errors also change together with the evolution of the system and increases in its accuracy?

The introduction of the article describes the premises for starting the discussion. The author refers to the works of selected authors and their own research which discuss the discrepancies between empirical statistics of errors in the navigation positioning systems and their theoretical values. The materials and methods section presents selected statistical distribution measures together with the interpretation of the histogram, probability-probability (P-P) plots, as well as differences between the empirical and theoretical cumulative distribution functions. In addition, the three types of statistical tests used in the research were described (Anderson-Darling, chi-square and Kolmogorov-Smirnov). The main research results are shown in the results section and discussed in the discussion section. The publication ends with conclusions and suggestions for further research.

This is the second article in a series of monothematic publications, the aim of which will be statistical distribution analysis of navigation positioning system errors.

## 2. Materials and Methods

### 2.1. Statistical Distribution Measures

Statistical testing to assess the consistency of empirical with theoretical distributions should be preceded by the calculation of specific distribution measures to determine their asymmetry, central tendency, concentration and dispersion. It should be noted that there is no specific set of distribution measures for specific analyses or processes in navigation or statistics. This selection should result from the statistical nature of the variable under investigation and the research aim. For example, for normal distributions of *φ* and *λ* errors using GPS, it makes sense to determine both the mean and the median. If these values are similar, this may indicate the empirical distribution fitting the normal distribution. However, for a 2D position error distribution (which exhibits an asymmetric chi-square distribution), it is not justified to determine both of these values as this distribution is asymmetric by its nature.

With this in mind, it is proposed to divide the assessment of the fit between error distributions in the navigation positioning systems and the theoretical distributions into two stages:

- Stage I: Calculation of selected statistical distribution measures: asymmetry (skewness), central tendency (arithmetic mean and median), concentration (kurtosis) and dispersion (range, standard deviation and variance).
- Stage II: Statistical testing using Anderson-Darling, chi-square and Kolmogorov-Smirnov tests.

Table 3 presents selected statistical distribution measures that will be used for empirical testing of 1D and 2D position errors. Their definitions, estimators, interpretations and properties are also given.

### 2.2. Analysis of the Histogram, P-P Plot, as Well as Differences Between the Empirical and Theoretical Cumulative Distribution Functions

A histogram is a very important element in assessing the distribution of the studied population. It is one of the graphic methods of representing the empirical distribution of a characteristic. It is made up of a series of rectangles placed on the axis of coordinates. These rectangles are, on the one hand, determined by the class interval values of the characteristic, while their height is determined by the number (or frequency, or possibly also probability density) of elements included in a given class interval. If the histogram shows the number of elements and not the probability density, then the interval widths should be equal.

In P-P plots, the empirical probability distribution function is plotted against the theoretical distribution. The observations are first sorted in descending order. The *i*-th observation is then plotted on one axis as $\frac{i}{n}$ (i.e., the value of the observed cumulative distribution) and the other axis as *F(x_i_)*, where *F(x_i_)* is the value of the theoretical probability distribution function for respective observation *x_i_*. If the theoretical cumulative distribution is a good approximation of the empirical distribution, then the points on the diagram should be close to the diagonal.

Regarding the idea behind them, the *F_n_*(*x*), *F*(*x*) and *F_n_(x)–F*(*x*) graphs are based on a comparison of empirical and theoretical distributions, similarly to P-P plots. They present both functions simultaneously or their differences.

### 2.3. Testing Statistical Distributions of Navigation Positioning System Errors

Testing statistical distributions of navigation positioning system errors is a key issue for assessing their distributions. This study tested the fit between 1D position errors (*φ* and *λ*) with the family of normal distributions. To this end, statistical hypotheses were verified, which means that any judgment on the population issued without detailed examination and verification was now tested. These allow determining whether the results obtained for the sample can be applied to the whole population [10].

In the literature review, it was noted that in statistical studies a large sample is considered to be a set consisting of at least 30 or 40 elements. Other samples are considered small. Furthermore, the sample size affects the choice of the type of statistical test. For example, the Shapiro-Wilk test, as confirmed by the experience of other authors, should be used for samples of less than 20 or 30 elements [11]. Another popular test, the Lilliefors test, is used to test the normality of distribution for samples of similar size to the Shapiro-Wilk test [12]. Tests such as the Cramér-von Mises test or the D’Agostino-Pearson test are used for statistical studies with large samples [13,14,15,16]. With this multitude of statistical tests, it was decided to choose the three most frequently used tests for large samples: Anderson-Darling, chi-square and Kolmogorov-Smirnov [17,18,19].

As in the statistical analysis, since it was planned to use records from a navigation positioning system ranging from several hundred thousand to over two million measurements, it became necessary to determine the appropriate sample size [20,21,22]. Based on the literature [23,24,25], to obtain the desired test power (0.8) at a significance level of 5% for the most popular statistical distributions, such as log-normal, normal, Weibull, etc., the statistical hypotheses should be tested for a sample size of about 1000 elements.

The approach to statistical testing presented above is based on a well-known statistical research theory. However, navigation positioning systems have a specific feature that distinguishes them from other measurement systems. This feature is the Position Random Walk (PRW). Its essence lies in the position coordinates “*walking*” around the reference coordinates. This issue has been described in detail in [26]. In this publication, a detailed analysis of this phenomenon was presented with the need to ensure a representative sample size highlighted based on empirical research. In addition, it shows that it is only selecting a representative sample size and 1000 measurements should be randomly drawn from this sample for statistical testing.

### 2.4. Statistical Tests Used in Research

The following statistical tests were used in the research:

- Anderson-Darling test: This test is based on the Cramér-von Mises weighted distance between the empirical *F_n_*(*x*) and theoretical *F*(*x*) distributions with weights corresponding to the inverse of the empirical distribution variance (note that *F_n_*(*x*) has a binomial distribution) [27]:

(2) $$d\left(F_{n},F\right)=n\int_{-\infty }^{\infty }\frac{{\left(F_{n}\left(x\right)-F\left(x\right)\right)}^{2}}{F\left(x\right)\left(1-F\left(x\right)\right)}dF\left(x\right)$$

Test statistics based on the above distance for a simple random sample *x_i_* may be written as:

(3) $$A^{2}=-n-S$$

where:

(4) $$S=\sum_{i=1}^{n}\frac{2i-1}{n}\left[\ln\left(F\left(x_{i}\right)\right)+\ln\left(1-F\left(x_{n+1-i}\right)\right)\right]$$

- Chi-square test: This test is based on the *χ*^2^ statistic [28]:
  (5) $$\chi^{2}=\sum_{i=1}^{n}\frac{{\left(O_{i}-E_{i}\right)}^{2}}{E_{i}}$$
  which, for a true zero hypothesis, has an asymptotic distribution *χ*^2^. The *E_i_* symbol indicates the expected number of observations in the *i* class and *O_i_* stands for the actual number of observations in the *i* class.

- Kolmogorov-Smirnov test: The test is based on the supremum distance between the empirical *F_n_*(*x*) and theoretical *F*(*x*) distribution functions [29,30]:

(6) $$d\left(F_{n},F\right)=\underset{x}{\sup}\left|F_{n}\left(x\right)-F\left(x\right)\right|$$

Test statistics based on the above distance consist of counting the maximum module of probability distribution difference at the empirical distribution function step points:

(7) $$D=\underset{x_{i}}{\max}\left|F_{n}\left(x_{i}\right)-F\left(x_{i}\right)\right|$$

### 2.5. Description of DGPS and EGNOS Measurement Campaigns

Studies of the position determination accuracy of the DGPS and EGNOS systems have been conducted in Poland for many years [31,32,33]. Due to the changing values of GPS position errors, which resulted in the increasing accuracy of DGPS and EGNOS augmentation systems, such research were conducted regularly in the years 2006 and 2014. The paper analyses two long-term measurement campaigns:

- The first measurement campaign took place in March 2006, in Gdynia (Poland). During this campaign, 2’187’842 fixes for DGPS and 1’774’705 fixes for EGNOS were recorded respectively with a recording frequency of 1 Hz.
- The second measurement campaign took place in May 2014, in Gdynia (Poland). During this campaign, 951’698 fixes for DGPS and 927’553 fixes for EGNOS were recorded respectively with a recording frequency of 1 Hz.

**Figure 3:**
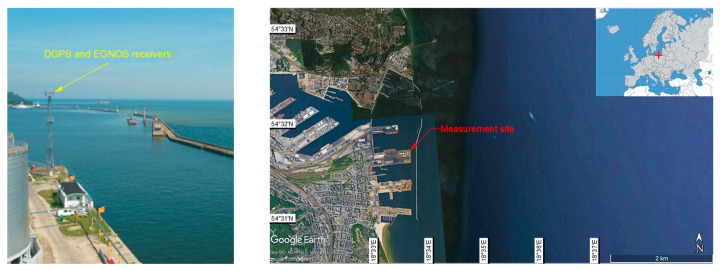
Measurement site-the radio beacon in the port of Gdynia.

Studies of both measurement campaigns included the installation of the DGPS and EGNOS receivers always in the same place-on the radio beacon in the port of Gdynia (Figure 3). It was a reference point with ellipsoidal coordinates amounting to: *φ* = 54°31.756087′ N, *λ* = 18°33.574138′ E and *h* = 68.070 m. During the measurement campaigns, typical DGPS (Leica MX9212 + MX51R) and EGNOS (Septentrio PolaRx2e) receivers with the possibility of saving data in the form of National Marine Electronics Association (NMEA) GGA messages were used. These measurements yielded text files with position coordinates which were compliant with the data transmission protocol described above. They contain geographic coordinates of designated points presented in angular (curvilinear) measure. To determine individual measurement errors, they were projected from the surface of the World Geodetic System 1984 (WGS 84) ellipsoid to a flat surface using Gauss-Krüger projection.

### 2.6. Research Assumptions

Basic assumptions for research and numerical analyses were as follows:

- Preliminary analyses carried out in [26] showed that a representative sample for DGPS and EGNOS systems should consist of about 900’000 measurements. Only with this sample size, 1D and 2D position errors are representative. Therefore, each of the analysed campaigns was shortened so that all sessions consist of the same number of measurements (900’000 fixes).
- Analysis of the campaigns (DGPS 2006, DGPS 2014, EGNOS 2006 and EGNOS 2014) with the same number of measurements makes it possible to compare their results and draw generalised conclusions.
- Selected statistical distribution measures (asymmetry, central tendency, concentration and dispersion) were determined for the same sample size (900’000 fixes).
- For statistical testing of fit between empirical distributions of 1D position errors (*φ* and *λ*) with the normal distribution, 1000 measurements were randomly selected and subjected to Anderson-Darling, chi-square and Kolmogorov-Smirnov tests.
- In comparative analyses of empirical distributions (1D and 2D position errors), the most frequently used theoretical distributions were used: Beta, Cauchy, chi-square, exponential, gamma, Laplace, logistic, lognormal, normal, Pareto, Rayleigh, Student’s and Weibull.
- Two values were used to assess position accuracy: the 2DRMS(2D) value, which was determined for the entire population (900’000 fixes) based on the relationship:

(8) $$2DRMS(2D)=2\sqrt{s_{\varphi }{}^{2}+s_{\lambda }{}^{2}}$$

where:

*s_φ_*—standard deviation of geodetic (geographic) latitude,
*s_λ_*—standard deviation of geodetic (geographic) longitude,

and the R95 value, which was determined by sorting the data from the lowest to the highest value.

- Easy Fit software was used for the analyses. To evaluate the fit of empirical with theoretical distributions, a significance level of 5% was assumed. The rankings of the best fit distributions were created based on the Kolmogorov-Smirnov statistic (*D*).
- Mathcad software was used to calculate the values of 2DRMS(2D) and R95(2D) and plot graphs of the position error distribution.

## 3. Results

### 3.1. DGPS 2006 Results

The study started with analyses of *φ* and *λ* error distributions assessed individually. Table 4 presents the results of statistical analysis and tests of *φ* and *λ* errors determined using the DGPS system in 2006. These include the evaluation of selected distribution measures and the results of testing the statistical fit of *φ* and *λ* errors with the normal distribution.

Next, in Table 5 the fit between empirical data of *φ* and *λ* errors and distributions other than the normal distribution for the DGPS system in 2006 was assessed.

Statistical analysis of *φ* and *λ* errors presented in Table 4 and Table 5 allows for the following conclusions:

- Central tendency measures: The mean values of *φ* and *λ* errors are very close to zero, which is indicative of a symmetrical distribution of 1D position errors in the N-S and E-W directions.
- Dispersion measures: The dispersion of *φ* and *λ* errors (*s*) is similar, with a similar range value, which indicates that the use of circular measures (2DRMS) of 2D position error is justified.
- Skewness: The latitude and longitude errors exhibit a weak asymmetry, thus both distributions can be considered to be symmetrical.
- Kurtosis: The latitude and longitude errors are leptokurtic (*Kurt* > 0), which means that they are more concentrated around the mean value than the normal distribution would suggest.
- Statistical testing: All tests have shown that *λ* error fits the normal distribution. The inverse relationship can be observed for *φ* error.
- Fit: Statistical distributions that fit empirical data best are beta (*φ* error) and lognormal (*λ* error) distributions. These distributions exhibit a much better fit to empirical data than the normal distribution.

Similar analyses were carried out with respect to the 2D position error. Their results are presented in Table 6 and Table 7.

Table 6 and Table 7 allows for the following conclusions:

- Please note that there are no outliers in the measurements under analysis, which indicates the high quality of the positioning services provided by the DGPS system.
- The 2DRMS and R95 values are similar, which confirms that the *φ* and *λ* error distributions have similar distributions.
- The distribution of 2D position error is, by its nature, asymmetrical, hence the best fit distributions include: beta, gamma, lognormal, Rayleigh and Weibull distributions.

### 3.2. DGPS 2014 Results

Similarly to the 2006 measurements, the results of the 2014 campaign analysis are presented in an identical tabular form in Table 8, Table 9, Table 10 and Table 11 below.

Statistical analysis of *φ* and *λ* errors presented in Table 8 and Table 9 allows for the following conclusions:

- Central tendency measures: The mean values of *φ* and *λ* errors are very close to zero, which is indicative of a symmetrical distribution of 1D position errors in the N-S and E-W directions.
- Dispersion measures: The latitude error has a dispersion (*s*) of about one and a half times higher than longitude error with a similar range value.
- Skewness: The latitude and longitude errors exhibit a very weak asymmetry, thus both distributions can be considered to be symmetrical.
- Kurtosis: The latitude and longitude errors are leptokurtic (*Kurt* > 0), which means that they are more concentrated around the mean value than the normal distribution would suggest. The very high concentration of 1D position errors represented by the kurtosis value [*Kurt*(*φ*) = 6.204 and *Kurt*(*λ*) = 12.956] needs emphasis. Compared to the 2006 measurement campaign, the system has significantly increased this parameter, which results in an increase in 2D position accuracy.
- Statistical testing: The Anderson-Darling and Kolmogorov-Smirnov tests have shown that *φ* error fits the normal distribution. However, all tests were rejected for *λ* error.
- Fit: The statistical distribution that fits empirical data best is the logistic distribution (*φ* and *λ* errors).

Similar analyses were carried out with respect to the 2D position error. Their results are presented in Table 10 and Table 11.

Table 10 and Table 11 allows for the following conclusions:

- Please note that there are no outliers in the measurements under analysis, which indicates the high quality of the positioning services provided by the DGPS system.
- The values of 2DRMS and R95 are similar. Moreover, the values of 2DRMS and R95 are below 1 m, which proves the very good accuracy of the system.
- The figure of the 2D position error distribution may suggest that the empirical distribution has a “*linear trend*”. However, this is not the case, because less than 0.17% (1496 fixes) of the studied population have an error greater than or equal to 2 m. Therefore, they can be considered as outliers.
- The distribution of 2D position error is, by its nature, asymmetrical, hence the best fit distributions include: beta, gamma, lognormal, Rayleigh and Weibull distributions.

### 3.3. EGNOS 2006 Results

Two EGNOS measurement campaigns were tested from two different implementation phases. The 2006 campaign dates from the period when the system was not fully operational [it was then in the initial operational capability (IOC) phase]. This can be interpreted as a period in which the system was not fully stable and there may have been some position errors classified as gross. However, the 2014 campaign was made in the Full Operational Capability (FOC). Table 12 presents the results of statistical analysis and tests of *φ* and *λ* errors determined using the EGNOS system in 2006. These include the evaluation of selected distribution measures and the results of testing the statistical fit of *φ* and *λ* errors with the normal distribution.

Studies have shown a very wide range of both *φ* (321.739 m) and *λ* (161.565 m) errors. Therefore, data represented by the kurtosis for *φ* and *λ* errors were very concentrated, although the average values of both variables are close to zero. Skewness calculated for λ error also reached a high value (1.410).

**Figure 4:**
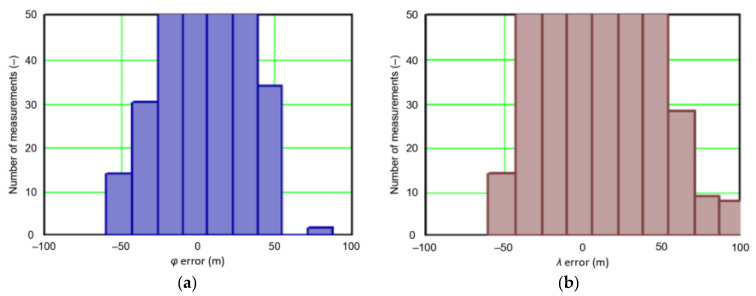
Histograms of *φ* (**a**) and *λ* (**b**) errors using the EGNOS system in 2006. The chart was cut to show the number of outliers.

In order to determine the numerical scale of outlier measurements that caused this anomaly, Figure 4 presents histograms of *φ* and *λ* errors to make the number of outliers visible.

Figure 4 shows that both for *φ* and *λ* errors outliers even by −60–60 m were quite frequent (more than 10 fixes). There is no doubt that the assessment of the statistical distribution of position errors from the EGNOS 2006 measurement campaign cannot be considered representative and no general conclusions can be drawn from it. Predictably, statistical testing of the fit between empirical data of *φ* and *λ* errors with the normal distribution showed a lack of fit.

Despite the anomalies identified in this campaign resulting from the status of the system (IOC), the EGNOS system was tested in the same way as the DGPS system. The results are presented in Table 13, Table 14 and Table 15.

Results presented in Table 13 indicate that the Cauchy distribution is the best fit for *φ* and *λ* errors.

Similar to the DGPS system, the analysis was carried out in relation to the 2D position error. The results are presented in Table 14 and Table 15.

From Table 14 and Table 15, it follows that a considerable number of outliers and the lack of fit between the errors and the normal distribution caused the values of 2DRMS and R95 to differ significantly. The distributions being the best fit for the EGNOS 2006 2D position error are: beta, exponential, gamma, lognormal and Weibull distributions.

### 3.4. EGNOS 2014 Results

The measurements of the EGNOS system carried out in 2014, which are analysed in this subsection, should be considered fully representative, since in 2014 the system operated in FOC mode. Table 16, Table 17, Table 18 and Table 19 present the results of statistical analyses. The method used was identical as for the DGPS 2006 and 2014 studies.

Statistical analysis of *φ* and *λ* errors presented in Table 16 and Table 17 allows for the following conclusions:

- Central tendency measures: The mean values of *φ* and *λ* errors are very close to zero, which is indicative of a symmetrical distribution of 1D position errors in the N-S and E-W directions.
- Dispersion measures: The dispersion of *φ* error (*s*) and the range value are almost twice the value for *λ* error.
- Skewness: The latitude error exhibits significant skewness to the right, whereas the longitude error exhibits slight skewness to the left.
- Kurtosis: The latitude and longitude errors are leptokurtic (*Kurt* > 0), which means that they are more concentrated around the mean value than the normal distribution would suggest.
- Statistical testing: The Anderson-Darling and Kolmogorov-Smirnov tests have shown that *λ* error fits the normal distribution. However, all tests were rejected for *φ* error.
- Fit: Statistical distributions that fit empirical data best are lognormal (*φ* error) and logistic (*λ* error) distributions. These distributions exhibit a much better fit to empirical data than the normal distribution.

Similar to the DGPS system, the analysis was carried out in relation to the 2D position error. The results are presented in Table 18 and Table 19.

Table 18 and Table 19 allows for the following conclusions:

- Please note that there are no outliers in the measurements under analysis, which indicates the high quality of the positioning services provided by the EGNOS system.
- The values of 2DRMS and R95 are similar. Moreover, the values of 2DRMS and R95 are below 1 m, which proves the very good accuracy of the system.
- The distribution of 2D position error is, by its nature, asymmetrical, hence the best fit distributions include: beta, gamma, lognormal, Rayleigh and Weibull distributions.

## 4. Discussion

In order to assess which of the statistical distributions are the best fit for empirical data of DGPS and EGNOS systems, Table 20 and Table 21 summarise the analyses and studies carried out. Points (1–10) were assigned to individual distributions to allow the selection of the best fit. The distributions being the best fit were assigned 10 points.

The use of distribution gradations (from the best to the worst fit) and assigning points to them made it possible to determine those distributions which present the best fit in three categories:

(1) Universal distribution where all the results from Table 20 and Table 21 were taken into account for evaluation. Both concerning 1D and 2D position errors.
(2) Best fit 1D position error distribution where the fit results for 1D errors were analysed from the following measurement campaign: DGPS 2006, DGPS 2014 and EGNOS 2014.
(3) Best fit 2D position error distribution where the fit results for 2D error were analysed from the following measurement campaign: DGPS 2006, DGPS 2014 and EGNOS 2014.

In the analyses in points 2 and 3, the results of the EGNOS 2006 measurement campaign were omitted due to its low representativeness. Cumulative results are presented in Table 22.

The results presented in Table 22 indicate that:

- The universality of the lognormal distribution which approximates both 1D and 2D position errors.
- Beta, gamma, logistic and Weibull distributions fit almost as well as the lognormal distribution.
- The normal distribution, commonly used for analysing navigation positioning system errors, is only suitable for 1D applications.
- The chi-square distribution, which is often recommended for position error analysis (especially 2D), shows no significant similarity to empirical data obtained from navigation positioning systems.

## 5. Conclusions

The Gauss distribution is commonly used to present results of accuracy analyses for the position determination by navigation systems. Due to the simplicity of calculations, the special features of standard deviation, as well as the intuitive perception of randomness in statistics to which this distribution corresponds, it is commonly used in science. It should be noted however that the necessary conditions for a random variable to be normally distributed include the independence of measurements and identical conditions of their realisation, which is not the case in the iterative method of determining successive positions, the filtration of coordinates or the dependence of the position error on meteorological conditions. The consistency of *φ* and *λ* errors was tested on DGPS and EGNOS systems. For each of the systems, the analyses used two measurement campaigns from 2006 and 2014.

Studies of DGPS (2006 and 2014) and EGNOS (2014) systems confirmed that *φ* and *λ* errors alternately fit the normal distribution, but also showed that the normal distribution is not an optimal statistical distribution to describe the navigation positioning system errors. The distributions that describe positioning system errors more accurately include: beta, gamma, logistic, lognormal and Weibull distributions. The results of the EGNOS 2006 measurement campaign cannot be considered representative and no general conclusions can be drawn from it. This is due to the fact that the EGNOS system was then in the IOC phase, hence numerous position errors classified as gross have appeared during the measurements (Figure 4). The research proved that in order to reliably determine navigation positioning system errors, statistical analyses should be performed using various distributions (by selecting the best one) for a representative sample size.

This is the second article in a series of monothematic publications [26], the aim of which will be statistical distribution analysis of navigation positioning system errors. One of the next research issues that has not been studied in this article will be to determine the impact of GNSS errors (ionospheric and tropospheric effects, multipath, noise, etc.) that influence the consistency of the empirical distributions of navigation positioning system errors with theoretical distributions.

## Figures and Tables

**Table 1:** Statistics of position errors of Samsung Galaxy phones during the dynamic measurement campaign. Own study based on: [8].

Statistics of Position Error	Samsung Galaxy					
	Y	S3 Mini	S4	S5	S6	S7
Number of measurements	6041	3410	10'950	10'939	10'906	10'926
2DRMS(2D)	9.47 m	5.23 m	6.59 m	6.72 m	8.32 m	10.54 m
R95(2D)	6.84 m	3.67 m	5.80 m	5.77 m	9.38 m	9.62 m
RPE	−38.45%	−42.51%	−13.62%	−16.46%	11.30%	−9.56%

**Table 2:** Statistics of position errors of Samsung Galaxy phones during the 24 h stationary measurement campaign. Own study based on: [9].

Statistics of Position Error	Samsung Galaxy					
	Y	S3 Mini	S4	S5	S6	S7
Number of measurements	73'699	71'438	86'290	86'346	86'371	86'355
2DRMS(2D)	5.61 m	6.79 m	2.04 m	2.06 m	13.69 m	8.93 m
R95(2D)	4.93 m	3.76 m	1.65 m	1.76 m	12.64 m	8.39 m
RPE	−13.79%	−80.59%	−23.64%	−17.05%	−8.31%	−6.44%

**Table 3:** Selected statistical distribution measures, their definitions, estimators, interpretations and properties, used to study *φ* and *λ* error distributions (separately) of navigation positioning systems.

Distribution Measure	Estimator	Definition/Properties/Interpretation
Arithmetic mean (central tendency measure)	${\bar{x}}_{n}=\frac{x_{1}+x_{2}+\ldots +x_{n}}{n}=\frac{\sum_{i=1}^{n}x_{i}}{n}$ where: ${\bar{x}}_{n}$—arithmetic mean from the sample, *x_i_*—subsequent values of a given random variable in the sample, *n*—sample size.	Definition: Arithmetic mean—the sum of numbers divided by their number. Properties: The arithmetic mean of a sample is, regardless of the distribution, a consistent and unbiased estimator of the expected value of the distribution from which the sample was drawn. The arithmetic mean is sensitive to the skewness and outlier observations.
Median (central tendency measure)	If *n* is an even number, the median (*m*) is: $m=\frac{x_{\frac{n}{2}}+x_{\frac{n}{2}+1}}{2}$ when *n* is an odd number, it *m*: $m=\frac{x_{\frac{n}{2}+1}}{2}$	Definition: Median—characteristic in ordered series, with an equal number of observations found above and below it. Properties: It is a measure that is much more resistant to outliers than the arithmetic mean.
Range (dispersion measure)	Range(x)=max(x)−min(x) where: *Range*(*x*)—range, *max*(*x*)—maximum value of a given random variable in the sample, *min*(*x*)—minimum value of a given random variable in the sample.	Definition: Range—the difference between the maximum and minimum value. Properties: Distorted by outliers. Leaves most information out. Not algebraically defined.
Variance and standard deviation (dispersion measures)	Unbiased variance estimator (*s^2^*): $s^{2}=\frac{1}{n-1}\sum_{i=1}^{n}(x_{i}-{\bar{x}}_{n}{)}^{2}$ Standard deviation of the sample (*s*): $s=\sqrt{\frac{{\sum_{i=1}^{n}\left(x_{i}-{\bar{x}}_{n}\right)}^{2}}{n-1}}$	Definition: Variance—the arithmetic mean of the squares of the deviations (differences) between the indvidual values of a characteristic and the expected value. Standard deviation—square root of the variance. Properties: Easy to calculate, they are defined algebraically. They take into account all values of characteristic variants. They are strongly influenced by outliers. Distortion in the case of skewed distributions. Difficult to compare for varied values.
Skewness (asymmetry measure)	$G=\frac{\frac{1}{n}\sum_{i=1}^{n}(x_{i}-{\bar{x}}_{n}{)}^{3}}{s^{3}}$ where: *G*—skewness.	Definition: Skewness—a measure of distribution’s asymmetry. Properties: It illustrates to what extent the arithmetic mean reflects the actual central tendency of the distribution. If its value is high, the arithmetic mean does not properly reflect the most typical measured value. In this case, the existence of outliers in the distribution may be suspected. As a result, data need correction of the application of non-parametric tests. This is very important when assessing the symmetry of a variable’s distribution. It determines the degree and direction of asymmetry, with a negative value standing for distribution skewed to the left and a positive value standing for distribution skewed to the right. Interpretation: |G|∈〈0,0.4)—very weak asymmetry, almost symmetrical distribution. |G|∈〈0.4,0.8)—weak asymmetry. |G|∈〈0.8,1.2)—moderate asymmetry. |G|∈〈1.2,1.6)—strong asymmetry. |G|∈〈1.6,∞)—very strong asymmetry.
Kurtosis (concentration measure)	$Kurt=\frac{n\left(n+1\right)}{\left(n-1\right)\left(n-2\right)\left(n-3\right)}\sum_{i=1}^{n}{\left(\frac{x_{i}-{\bar{x}}_{n}}{s}\right)}^{4}-\frac{3{\left(n-1\right)}^{2}}{\left(n-2\right)\left(n-3\right)}$ where: *Kurt*—sample kurtosis.	Definition: Kurtosis—a measure of distribution’s flattening. Properties: It measures whether the distribution is “*peaky*”. If the kurtosis value is clearly different from zero, then the distribution is either flatter or more pointed than the normal distribution. The kurtosis for a normal distribution is zero. Interpretation: Mesokurtic distributions (*Kurt* = 0 for a normal distribution)—the kurtosis value is zero, the flattening distribution is similar to the flattening of a normal distribution (for which the kurtosis is exactly zero). Leptokurtic distributions (*Kurt* > 0 for a slender distribution)—the kurtosis value is positive, the characteristic values are more concentrated than in the normal distribution. Platykurtic distributions (*Kurt* < 0 for a flattened distribution)—the kurtosis value is negative, the characteristic values are less concentrated than in the normal distribution.

**Table 4:** Statistical analysis of distribution measures and statistical tests of *φ* and *λ* errors using the DGPS system in 2006.

Distribution Measure	*φ* Error	λ Error	Probability Density Function for *φ* Error		Probability Density Function for *λ* Error	
Sample size	900’000		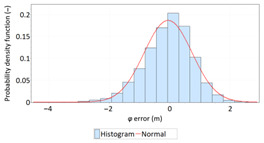		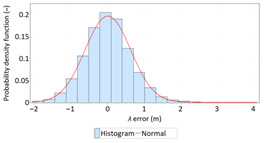	
Arithmetic mean	−0.058 m	0.005 m				
Median	0.003 m	−0.007 m				
Range	7.395 m	6.167 m				
Variance	0.626 m	0.394 m				
Standard deviation	0.791 m	0.628 m				
Skewness	−0.435	0.208				
Kurtosis	0.739	0.684				
			Anderson-Darling	Reject	Anderson-Darling	No reject
			Chi-square	Reject	Chi-square	No reject
			Kolmogorov-Smirnov	Reject	Kolmogorov-Smirnov	No reject

**Table 5:** Analysis of fit between empirical data of *φ* and *λ* errors and distributions other than the normal distribution for the DGPS system in 2006.

Best Fit Distribution for *φ* Error		Best Fit Distribution for *λ* Error	
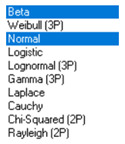	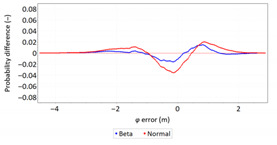	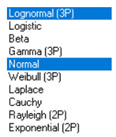	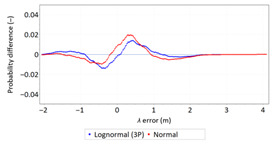

**Table 6:** Statistical analysis of distribution measures of 2D position error using the DGPS system in 2006.

Distribution Measure	2D Position Error	Probability Density Function for 2D Position Error	2D Position Error Distribution
Sample size	900’000	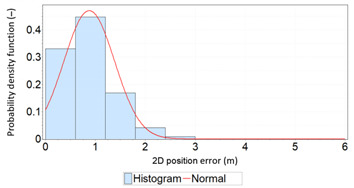	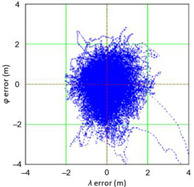
Arithmetic mean	0.875 m		
Median	0.802 m		
Range	5.993 m		
Variance	0.258 m		
Standard deviation	0.508 m		
Skewness	1.192		
Kurtosis	3.207		
2DRMS(2D)	2.013 m		
R95(2D)	1.815 m		

**Table 7:** Analysis of fit between empirical data of 2D position error and distributions other than the normal distribution for the DGPS system in 2006.

Best Fit Distribution for 2D Position Error ^1^	
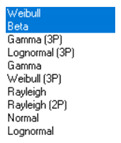	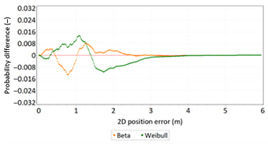

^1^ The distribution parameters are generally known and will not be described in detail.

**Table 8:** Statistical analysis of distribution measures and statistical tests of *φ* and *λ* errors using the DGPS system in 2014.

Distribution Measure	*φ* Error	λ Error	Probability Density Function for *φ* Error		Probability Density Function for *λ* Error	
Sample size	900’000		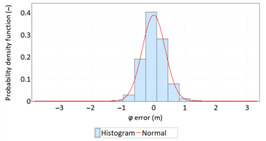		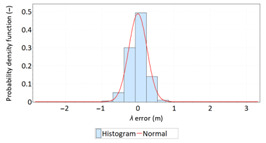	
Arithmetic mean	−0.001 m	−0.001 m				
Median	−0.013 m	0.010 m				
Range	7.176 m	6.153 m				
Variance	0.135 m	0.063 m				
Standard deviation	0.368 m	0.251 m				
Skewness	−0.114	0.216				
Kurtosis	6.204	12.956				
			Anderson-Darling	No reject	Anderson-Darling	Reject
			Chi-square	Reject	Chi-square	Reject
			Kolmogorov-Smirnov	No reject	Kolmogorov-Smirnov	Reject

**Table 9:** Analysis of fit between empirical data of *φ* and *λ* errors and distributions other than the normal distribution for the DGPS system in 2014.

Best Fit Distribution for *φ* Error		Best Fit Distribution for *λ* Error	
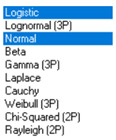	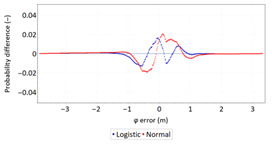	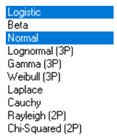	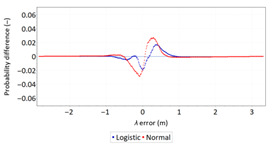

**Table 10:** Statistical analysis of distribution measures of 2D position error using the DGPS system in 2014.

Distribution Measure	2D Position Error	Probability Density Function for 2D Position Error	2D Position Error Distribution
Sample size	900’000	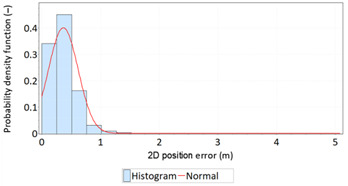	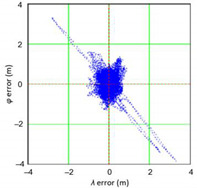
Arithmetic mean	0.367 m		
Median	0.330 m		
Range	5.076 m		
Variance	0.064 m		
Standard deviation	0.252 m		
Skewness	4.513		
Kurtosis	54.192		
2DRMS(2D)	0.885 m		
R95(2D)	0.748 m		

**Table 11:** Analysis of fit between empirical data of 2D position error and distributions other than the normal distribution for the DGPS system in 2014.

Best Fit Distribution For 2D Position Error	
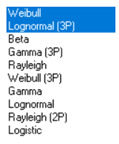	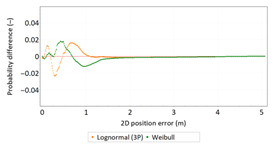

**Table 12:** Statistical analysis of distribution measures and statistical tests of *φ* and *λ* errors using the EGNOS system in 2006.

Distribution Measure	*φ* Error	λ Error	Probability Density Function for *φ* Error		Probability Density Function for *λ* Error	
Sample size	900’000		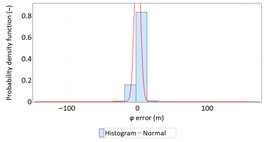		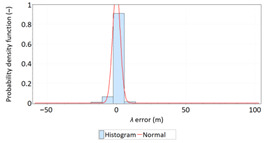	
Arithmetic mean	−0.297 m	0.026 m				
Median	0.194 m	0.111 m				
Range	321.739 m	161.565 m				
Variance	18.051 m	7.509 m				
Standard deviation	4.249 m	2.740 m				
Skewness	−0.567	1.410				
Kurtosis	44.819	68.963				
			Anderson-Darling	Reject	Anderson-Darling	Reject
			Chi-square	Reject	Chi-square	Reject
			Kolmogorov-Smirnov	Reject	Kolmogorov-Smirnov	Reject

**Table 13:** Analysis of fit between empirical data of *φ* and *λ* errors and distributions other than the normal distribution for the EGNOS system in 2006.

Best Fit Distribution for *φ* Error		Best Fit Distribution for *λ* Error	
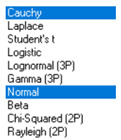	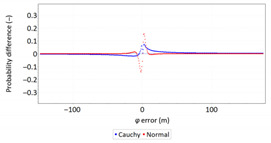	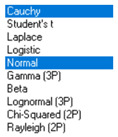	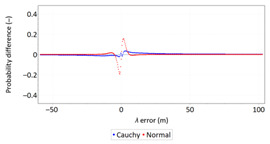

**Table 14:** Statistical analysis of distribution measures of 2D position error using the EGNOS system in 2006.

Distribution Measure	2D Position Error	Probability Density Function for 2D Position Error	2D Position Error Distribution
Sample size	900’000	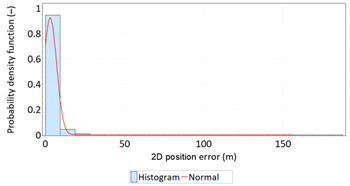	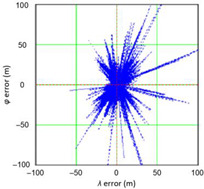
Arithmetic mean	3.033 m		
Median	1.823 m		
Range	187.687 m		
Variance	16.447 m		
Standard deviation	4.056 m		
Skewness	5.633		
Kurtosis	72.045		
2DRMS(2D)	8.390 m		
R95(2D)	9.984 m		

**Table 15:** Analysis of fit between empirical data of 2D position error and distributions other than the normal distribution for the EGNOS system in 2006.

Best Fit Distribution for 2D Position Error	
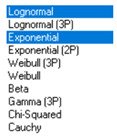	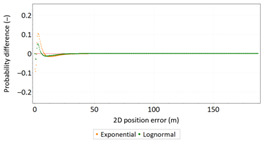

**Table 16:** Statistical analysis of distribution measures and statistical tests of *φ* and *λ* errors using the EGNOS system in 2014.

Distribution Measure	*φ* Error	λ Error	Probability Density Function for *φ* Error		Probability Density Function for *λ* Error	
Sample size	900’000		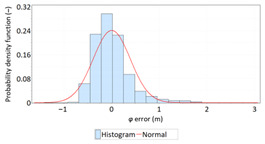		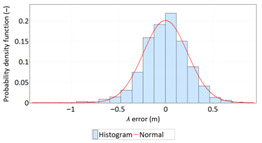	
Arithmetic mean	0.005 m	−0.004 m				
Median	−0.057 m	−0.083 m				
Range	4.689 m	2.360 m				
Variance	0.151 m	0.055 m				
Standard deviation	0.388 m	0.235 m				
Skewness	1.579	−0.204				
Kurtosis	4.375	0.983				
			Anderson-Darling	Reject	Anderson-Darling	No reject
			Chi-square	Reject	Chi-square	Reject
			Kolmogorov-Smirnov	Reject	Kolmogorov-Smirnov	No reject

**Table 17:** Analysis of fit between empirical data of *φ* and *λ* errors and distributions other than the normal distribution for the EGNOS system in 2014.

Best Fit Distribution for *φ* Error		Best Fit Distribution for *λ* Error	
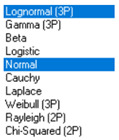	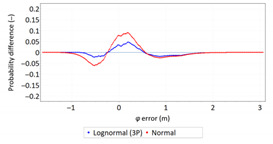	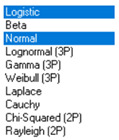	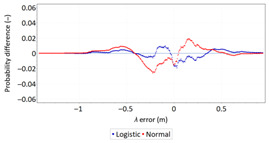

**Table 18:** Statistical analysis of distribution measures of 2D position error using the EGNOS system in 2014.

Distribution Measure	2D Position Error	Probability Density Function for 2D Position Error	2D Position Error Distribution
Sample size	900’000	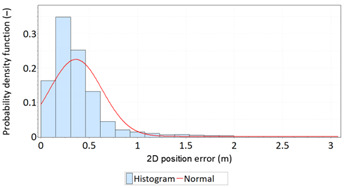	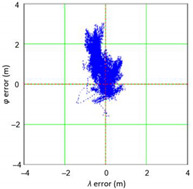
Arithmetic mean	0.363 m		
Median	0.304 m		
Range	3.071 m		
Variance	0.074 m		
Standard deviation	0.273 m		
Skewness	2.548		
Kurtosis	9.920		
2DRMS(2D)	0.901 m		
R95(2D)	0.854 m		

**Table 19:** Analysis of fit between empirical data of 2D position error and distributions other than the normal distribution for the EGNOS system in 2014.

Best Fit Distribution for 2D Position Error	
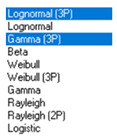	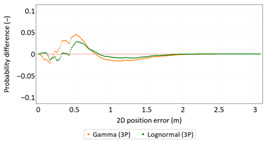

**Table 20:** Statistical distributions being the best fit for empirical data from DGPS 2006 and 2014 measurement campaigns.

Ranking of the Best Fit Distributions											
DGPS 2006						DGPS 2014					
*φ* Error		*λ* Error		2D Position Error		*φ* Error		*λ* Error		2D Position Error	
1. Beta	10 pt	1. Lognormal (3P)	10 pt	1. Weibull	10 pt	1. Logistic	10 pt	1. Logistic	10 pt	1. Weibull	10 pt
2. Weibull (3P)	9 pt	2. Logistic	9 pt	2. Beta	9 pt	2. Lognormal (3P)	9 pt	2. Beta	9 pt	2. Lognormal (3P)	9 pt
3. Normal	8 pt	3. Beta	8 pt	3. Gamma (3P)	8 pt	3. Normal	8 pt	3. Normal	8 pt	3. Beta	8 pt
4. Logistic	7 pt	4. Gamma (3P)	7 pt	4. Lognormal (3P)	7 pt	4. Beta	7 pt	4. Lognormal (3P)	7 pt	4. Gamma (3P)	7 pt
5. Lognormal (3P)	6 pt	5. Normal	6 pt	5. Gamma	6 pt	5. Gamma (3P)	6 pt	5. Gamma (3P)	6 pt	5. Rayleigh	6 pt
6. Gamma (3P)	5 pt	6. Weibull (3P)	5 pt	6. Weibull (3P)	5 pt	6. Laplace	5 pt	6. Weibull (3P)	5 pt	6. Weibull (3P)	5 pt
7. Laplace	4 pt	7. Laplace	4 pt	7. Rayleigh	4 pt	7. Cauchy	4 pt	7. Laplace	4 pt	7. Gamma	4 pt
8. Cauchy	3 pt	8. Cauchy	3 pt	8. Rayleigh (2P)	3 pt	8. Weibull (3P)	3 pt	8. Cauchy	3 pt	8. Lognormal	3 pt
9. Chi-square (2P)	2 pt	9. Rayleigh (2P)	2 pt	9. Normal	2 pt	9. Chi-square (2P)	2 pt	9. Rayleigh (2P)	2 pt	9. Rayleigh (2P)	2 pt
10. Rayleigh (2P)	1 pt	10. Expotential (2P)	1 pt	10. Lognormal	1 pt	10. Rayleigh (2P)	1 pt	10. Chi-square (2P)	1 pt	10. Logistic	1 pt

**Table 21:** Statistical distributions being the best fit for empirical data from EGNOS 2006 and 2014 measurement campaigns.

Ranking of the Best Fit Distributions											
EGNOS 2006						EGNOS 2006					
*φ* Error		*λ* Error		2D Position Error		*φ* Error		*λ* Error		2D Position Error	
1. Cauchy	10 pt	1. Cauchy	10 pt	1. Lognormal	10 pt	1. Lognormal (3P)	10 pt	1. Logistic	10 pt	1. Lognormal (3P)	10 pt
2. Laplace	9 pt	2. Student’s	9 pt	2. Lognormal (3P)	9 pt	2. Gamma (3P)	9 pt	2. Beta	9 pt	2. Lognormal	9 pt
3. Student’s	8 pt	3. Laplace	8 pt	3. Expotential	8 pt	3. Beta	8 pt	3. Normal	8 pt	3. Gamma (3P)	8 pt
4. Logistic	7 pt	4. Logistic	7 pt	4. Expotential (2P)	7 pt	4. Logistic	7 pt	4. Lognormal (3P)	7 pt	4. Beta	7 pt
5. Lognormal (3P)	6 pt	5. Normal	6 pt	5. Weibull (3P)	6 pt	5. Normal	6 pt	5. Gamma (3P)	6 pt	5. Weibull	6 pt
6. Gamma (3P)	5 pt	6. Gamma (3P)	5 pt	6. Weibull	5 pt	6. Cauchy	5 pt	6. Weibull (3P)	5 pt	6. Weibull (3P)	5 pt
7. Normal	4 pt	7. Beta	4 pt	7. Beta	4 pt	7. Laplace	4 pt	7. Laplace	4 pt	7. Gamma	4 pt
8. Beta	3 pt	8. Lognormal (3P)	3 pt	8. Gamma (3P)	3 pt	8. Weibull (3P)	3 pt	8. Cauchy	3 pt	8. Rayleigh	3 pt
9. Chi-square (2P)	2 pt	9. Chi-square (2P)	2 pt	9. Chi-square	2 pt	9. Rayleigh (2P)	2 pt	9. Chi-square (2P)	2 pt	9. Rayleigh (2P)	2 pt
10. Rayleigh (2P)	1 pt	10. Rayleigh (2P)	1 pt	10. Cauchy	1 pt	10. Chi-square (2P)	1 pt	10. Rayleigh (2P)	1 pt	10. Logistic	1 pt

**Table 22:** Statistical distributions which were the best fit for the measurement campaigns analysed depending on the position dimension (1D, 2D or 1D and 2D).

Ranking of the Best Fit Distributions					
1D Position Error		2D Position Error		1D+2D Position Errors	
1. Logistic	53 pt	1. Lognormal (3P)	26 pt	1. Lognormal (3P)	93 pt
2. Beta	51 pt	2. Weibull	26 pt	2. Beta	86 pt
3. Lognormal (3P)	49 pt	3. Beta	24 pt	3. Gamma (3P)	75 pt
4. Normal	44 pt	4. Gamma (3P)	23 pt	4. Logistic	69 pt
5. Gamma (3P)	39 pt	5. Weibull (3P)	15 pt	5. Normal	56 pt
6. Weibull (3P)	30 pt	6. Gamma	14 pt	6. Weibull (3P)	51 pt
7. Laplace	25 pt	7. Rayleigh	13 pt	7. Laplace	42 pt
8. Cauchy	21 pt	8. Lognormal	13 pt	8. Cauchy	42 pt
9. Rayleigh (2P)	9 pt	9. Rayleigh (2P)	7 pt	9. Weibull	31 pt
10. Chi-square (2P)	8 pt	10. Normal	2 pt	10. Lognormal	23 pt
